# Personality Dynamism and Academic Performance Among Boarders and Non-boarders Studying in a Medical University

**DOI:** 10.7759/cureus.5072

**Published:** 2019-07-03

**Authors:** Muhammad Sarfraz Khan, Abdur Rehman Malik, Adam Umair Ashraf Butt, Areeb Khalid, Shahzaib Maqbool, Hashim Khan, Muhammad Waqar Younas

**Affiliations:** 1 Internal Medicine: Gastroenterology, Rawalpindi Medical College, Rawalpindi, PAK; 2 Neurology, Rawalpindi Medical College, Rawalpindi, PAK; 3 Urology, Rawalpindi Medical College, Rawalpindi, PAK; 4 Surgery, Rawalpindi Medical College, Rawalpindi, PAK; 5 Pediatrics, Rawalpindi Medical College, Rawalpindi, PAK; 6 Cardiology, Rawalpindi Medical College, Rawalpindi, PAK; 7 Internal Medicine, Rawalpindi Medical College, Rawalpindi, PAK

**Keywords:** big five inventory, boarders, non-boarders, extraversion, agreeableness, conscientiousness, neuroticism, openness

## Abstract

Introduction

The personality of a human being consists of his behavior, cognition, emotional abilities, and interaction with his surroundings. The personality of an individual is modified by his ability for social perception, life experiences, and training. The pattern of study in which a person acquires education has a great influence on their personality.

Objective

To compare the personality traits and academic performance of boarders and non-boarders studying in a medical university.

Material and methods

It is a comparative, descriptive cross-sectional study done at Rawalpindi Medical University, Pakistan. The duration of the study was from January 2019 to April 2019. A questionnaire was randomly distributed among the students of Rawalpindi Medical University and filled under supervision. The questionnaire had two parts: (1) Academic performance information and (2) Big Five inventory (BFI-40). Only MBBS students of Rawalpindi Medical University were included. For statistical analysis, the independent t-test was applied using the Windows IBM Statistical Package for the Social Sciences Version 22 (SPSS, IBM Corp., Armonk, NY, US). The statistically significant value was taken as 0.05.

Results

Out of 300 questionnaires distributed, 287 were properly filled, giving a response rate of 95.6%. The Cronbach's alpha value was .750. The mean age was 20.87±1.344. There were 216 (75.1%) male and 71 (24.9%) females. One-hundred eighty-three (63.8%) were boarders and 104 (36.2%) were non-boarders. The mean scores of extraversion, agreeableness, and conscientiousness were higher for boarders while the mean scores of neuroticism and openness were higher for non-boarders. High average percentages in professional exams were common in non-boarders while boarders were taking more supplementary exams comparatively.

Conclusion

Self-discipline, surgency from external activities/situations, and getting along with others are common traits among boarders. On the other hand, non-boarders are more creative but emotionally unstable. The academic status of boarders is comparatively poor. Thus, the hostel administration should be particularly concerned about the activities of boarders, and parents should be aware of their child's academic status. Teachers should pay special attention to the character development of students.

## Introduction

The personality of a human being consists of his behavior, cognition, emotional abilities, and interaction with his surroundings [1]. The personality of an individual is modified by his ability of social perception, life experiences, and training. The pattern of study in which a person acquires education has a great influence on his personality [2]. The dynamics of personality are altered by environmental factors, and the difference in the personality of different individuals can predict the different lifestyles, learning habits, cognitive abilities, communication skills, and problem-solving skills [3]. It is a common saying that," a person is known by the company he keeps", which has been proven by the socialization theory of Judith Rich Harris. It states that an individual’s peer group has greater importance in the development of personality in adulthood than the parents' contribution [4]. Life experiences also have an impact on personality development; the accretion of experience from daily life activities enables persons to cope with different kinds of situations in the future. It all depends on an individual’s discernment of environmental stimuli and their response to unraveling them [5]. Personality is a complex concept, and many tests are available to determine it. The two main tests include objective tests and projective measures, for example, Big Five Inventory (BFI), Minnesota Multiphasic Personality Inventory (MMPI2), Rorschach Inkblot Test, Neurotic Personality Questionnaire KON-2006, or Eysenck’s Personality Questionnaire (EPQ-R). The usefulness of these tests in determining personality depends upon their reliability and validity [1]. In this study, we applied the BFI model to determine personality dynamics. The BFI-44 has been proven reliable in multiple languages [6].

According to BFI, personality is differentiated into five factors or traits. These include openness to experience (imaginativeness, broad-mindedness, and artistic sensibility), extraversion (activity and sociability), conscientiousness (dependability and will to achieve), agreeableness (reflecting likability and friendliness), and neuroticism. Different archetypes of behavior can be accurately foreseen by this model, but it cannot predict specific behavior [7].

An important aspect of the educational life of most students is a residency in educational institutes, which is also known as hostel life. Hostel life has many bearings on both the personality as well as the academic performance of the students. Conferring different studies, it was found that the boarding environment has significant effects on the student’s learning abilities, skills, emotional abilities, social life, and academic performance. Another consequence of boarding life is homesickness, which results in loss of concentration and lack of attention in daily activities [8-9]. While boarding schools cause many problems, such as emotional disturbance, adoption of filthy habits, and crushing family bonds, they have a positive impact on the development of the student’s personality [10-11]. The purpose of this research is to investigate the difference in the personality dynamics between boarders and non-boarders as well as the difference in their academic performance.

## Materials and methods

This is a descriptive, cross-sectional study done at Rawalpindi Medical University, Pakistan. The duration of the study was from January 2019 to April 2019. A questionnaire was randomly distributed among the students of Rawalpindi Medical University and filled under supervision. The questionnaire had two parts: (1) Academic performance information and (2) Big Five Inventory (BFI-44). The BFI-44 comprises 44 questions covering five personality traits: extraversion, agreeableness, conscientiousness, neuroticism, and openness. All the boarders living in university hostels were included in this study. For statistical analysis, the independent t-test was applied using IBM Statistical Package for the Social Sciences Version 22 (SPSS; IBM, Armonk, NY, US). A p-value of ≤0.05 was taken as significant.

## Results

Out of the 300 questionnaires distributed, 287 were properly filled, giving a response rate of 95.6%. The Cronbach’s alpha value was .750.

Demographic details

The mean age was 20.87±1.344. There were 216 (75.1%) male and 71 (24.9%) females while 183 (63.8%) were boarders and 104 (36.2%) were non-boarders. One-hundred twenty-two (42.5%) were living in Rawalpindi and Islamabad; however, 107 (37.2%) belonged to Central Punjab. Thirty-six (12.7%) had their hometowns in South Punjab while 22 (7.6%) were from other provinces of Pakistan. Taking Rawalpindi Medical University as a center, the mean of the approximate traveling time was 4.95 hr ± 7.48.

Personality dynamism

a) Boarders vs. Non-Boarders

It was found that the mean scores of extraversion, agreeableness, and conscientiousness were higher comparatively among boarders while the mean scores of neuroticism and openness were higher among non-boarders. Table 1 shows the mean scores in each personality trait.

**Table 1:** Big Five Inventory-44 mean scores; boarders vs non-boarders

Personality traits	NON-BOARDERS		BOARDERS	
	Mean	S.D	Mean	S.D
Extraversion:	8.22	5.125	8.29	5.773
Agreeableness:	10.82	5.019	11.24	3.99
Conscientiousness:	6.27	5.143	6.66	6.612
Neuroticism:	5.98	6.527	3.95	6.042
Openness:	20.47	3.852	19.57	4.362

b) Among All Academic Years of MBBS

**Figure 1:**
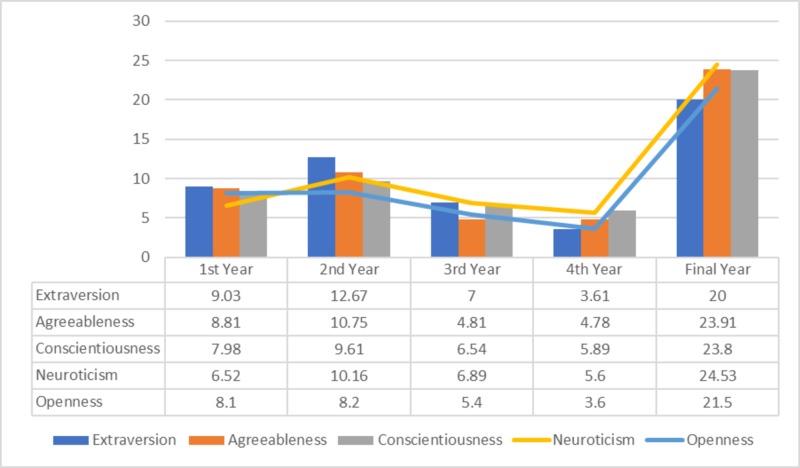
Big Five Inventory(BFI-44) mean scores in all academic years

Comparing the mean scores in all the academic years, it was found that final year students scored the highest in all the dynamics of personality (Figure 1).

Academic performance

a) Professional Exams Average Percentage Scores

The comparative analysis showed that only 76 (41.5%) boarders had n average percentage >70% while 57.6% non-boarders had an average percentage >70% in professional exams. The academic performance and boarding status of the student was significantly associated at p<0.001. The average percentage scores in professional exams are shown in Table 2.

**Table 2:** Average percentage scores boarders vs non-boarders

	Status		
%age	Boarders	Non-boarders	p-value
1st year^(a)^	78 (42.6%)	42 (40.8%)	p=0.140
61-70%	29 (15.7%)	2 (2.0%)	p<0.001
71-80%	63 (34.4%)	51 (49.0%)	p<0.001
>80%	13 (7.2%)	9 (8.2%)	p=0.133
(a) These students are in 1st year MBBS and haven’t appeared in any professional exams yet.			

b) Number of Supplementary Exams Taken

The mean number of supplementary exams was statistically different between boarders and non-boarders as shown by the independent samples t-test. Table 3 shows a comparison of supplementary exams between boarders and non-boarders.

**Table 3:** Number of supplementary exams taken by boarders vs non-boarders

	Status		
No. of supplementary exams	Boarder	Non-boarder	p-value
0	158 (86.1%)	102 (98%)	0.001
1	12 (6.6%)	2 (2%)	0.025
2	10 (5.7%)	0	0.033
3	3 (1.6%)	0	0.259

## Discussion

The study did not show a significant difference between boarders and non-boarders in the facets of extraversion, agreeableness, and conscientiousness. Even though the boarders scored marginally higher in these departments, the results are not significant enough to be considered reliable (p>0.05).

On the other hand, non-boarders had a significantly higher (p<0.05) association with neuroticism. These, in a way, show immaturity in the personalities of non-boarders due to their living in a somewhat sheltered environment. Openness was another field in which the results showed significantly higher scores for non-boarders. Tomar et al. also found neuroticism to be higher in non-boarders as compared to boarders [12].

Our results were similar to the findings obtained by Perveen in 2011. The study shows similar results, with non-boarders leading in openness. Neuroticism, on the other hand, was found to be higher in boarders [10]. A study conducted by Dr. Nayar in 2018 supported the finding that the facets that make up neuroticism (depression and anxiety.) were more commonly found in boarders [13]. Similarly, in another study, Singhvi et al. found that symptoms of neurotic depression were found to be significantly higher in hostel residents [14].

Marwaha et al. investigated the level of adjustment in boarders and non-boarders, and found out that the adjustment in the health, emotional, and home areas was significantly higher for non-boarders, with a higher overall adjustment as well [15]. This is in contrast to our results, which show neuroticism to be higher in non-boarders.

Academic performance was judged on the basis of average scores in Annual Professional Examinations as well as the number of time a student had to retake an exam. This study showed a very clear relationship showing non-boarders scoring significantly better (p<0.001) and taking fewer re-takes (p=0.009) as compared to boarders in our study.

Studies all over the world have shown similar results. An example of this is the study conducted by Khurshid et al. [16]. A study in India as well another one by Dambudzu came to similar conclusions that non-boarders were performing better academically when compared to non-boarders [17-18]. A possible factor for these results was investigated. Sleep deprivation is something that can lead to decreased scores in exams as found by a study by Dresler et al. [19]. A study in India came to the conclusion that a large number of boarders suffered from sleep deprivation, which ultimately led to a fall in academic performance [20].

Students’ academic gain is dependent upon multiple factors that include, but are not limited to, gender, age, teaching faculty, schooling, father/guardian social economic status, residential area, daily study hours, and accommodation as boarder or day scholar [21]. Thus, sometimes, being boarders or non-boarders may not key in at all in a student’s academic performance. Khan et al. conducted a study in Faisalabad, Pakistan, in which they compared the scores of students at the end of the first semester and then the Cumulative Grade Point Average (CGPA) at the end of the last. This is an excellent test to see whether living conditions affect academic performance over a long period of time. However, the study concluded that being a boarder or a non-boarder did not significantly affect their exam scores [22]. A study by Faisal et al. also found no significant difference between the academic performances of boarders and non-boarders [23].

In contrast to the above studies, a study in Kenya found the lack of parental contribution and support to be a major factor in the poor performance of non-boarders as compared to non-boarders [24]. Similar results were found by Grantham et al. [25].

Some other factors can also be responsible for the comparatively poor performance by non-boarders. These include a longer daily traveling time to and from college, which results in exhaustion and, consequently, degradation in performance. Bahadur et al., in a study in Rawalpindi as well as in Kenya, found this to be a significant factor that affects the performance of non-boarders [26-27].

The limitations of this study include the small sample size as well as the focus on a single college of a specific discipline, i.e., medicine. Further countrywide studies including institutions of different disciplines as well as different educational levels should be done in order to obtain more accurate results that can be generalized.

## Conclusions

Boarders have a higher tendency to display self-discipline, surgency from external activities/situations, and get along with others as compared to non-boarders. On the other hand, non-boarders are more creative but emotionally unstable. The academic status of boarders is comparatively poor. Thus, the hostel administration should be particularly concerned about the activities of boarders, and parents should be aware of their child's academic status. Teachers should pay special attention to the character development of students.
